# Physical multimorbidity and subjective cognitive complaints among adults in the United Kingdom: a cross-sectional community-based study

**DOI:** 10.1038/s41598-019-48894-8

**Published:** 2019-08-27

**Authors:** Louis Jacob, Josep Maria Haro, Ai Koyanagi

**Affiliations:** 1 Faculty of Medicine, University of Versailles Saint-Quentin-en-Yvelines, Montigny-le-Bretonneux, 78180 France; 2 Research and Development Unit, Parc Sanitari Sant Joan de Déu, CIBERSAM, Dr. Antoni Pujadas, 42, Sant Boi de Llobregat, Barcelona, 08830 Spain; 3 ICREA, Pg. Lluis Companys 23, Barcelona, 08010 Spain

**Keywords:** Epidemiology, Epidemiology, Risk factors

## Abstract

Our goal was to examine the association between physical multimorbidity and subjective cognitive complaints (SCC) using UK nationally representative cross-sectional community-based data, and to quantify the extent to which a broad range of mainly psychological and behavioral factors explain this relationship. Data from the 2007 Adult Psychiatric Morbidity Survey were analyzed [N = 7399 adults, mean (SD) age 46.3 (18.6) years, 48.6% men]. Multimorbidity was defined as ≥2 physical diseases. SCC included two different cognitive constructs: subjective concentration and memory complaints. Multivariable logistic regression and mediation analyses were conducted. Multimorbidity was associated with higher prevalence of subjective concentration (30.7% vs. 17.3%) and memory complaints (42.8% vs. 22.9%) compared to no multimorbidity. In the regression model adjusted for sociodemographics, multimorbidity was associated with subjective concentration (OR = 2.58; 95% CI = 2.25–2.96) and memory complaints (OR = 2.34; 95% CI = 2.08–2.62). Sleep problems, stressful life events and any anxiety disorder explained 21–23%, 20–22% and 14–15% of the multimorbidity-SCC association, respectively. Multimorbidity and SCC are highly co-morbid. The utility of SCC screening in identifying individuals at high risk for future cognitive decline among individuals with multimorbidity should be assessed.

## Introduction

Cognition is commonly defined as the ability to process information from the environment and the internal representation of this information via multiple mechanisms^1^. Cognition includes different cognitive processes such as attention, concentration, memory, reasoning, and decision-making. Subjective cognitive complaints (SCC) are everyday memory and related cognitive concerns expressed by people with or without objective evidence of cognitive impairment and are common in all age groups^2^. A nationally representative study of adults from the UK showed that almost one third of the population reported forgetfulness in the last month, and that more than 6% had forgotten something important in the last week^3^. It was further observed in another study conducted in the same country that the prevalence of subjective concentration complaints was between 7% and 10% in the general population^4^. Although there is controversy regarding the degree to which SCC and objective measures of cognition (e.g., assessed using standardized neuropsychological testing) correlate^2^, it is recognized that identifying SCC can provide a meaningful indicator of a person’s current cognitive functioning and may identify people at pronounced risk of future cognitive decline, even in the presence of normal baseline objective cognition^2^. Biological changes such as increases in white matter lesions, temporal atrophy, and altered cerebrospinal fluid biomarkers have also been observed in individuals with SCC^5–7^.

In recent years, there has been a growing interest in the relationship between multimorbidity and subjective cognitive function^8–10^. Multimorbidity, which is often defined as ≥2 chronic diseases in a single individual, is increasingly common, and has a significant impact on disability, quality of life, polypharmacy, premature mortality and health care costs^11^. Given that SCC is also known to substantially undermine quality of life^12^, and to be positively associated with mental disorders^13^ and all-cause mortality^14^, it is of vital importance to understand the co-occurrence of SCC and multimorbidity. Furthermore, several longitudinal studies have found that multimorbidity may precede cognitive decline as assessed by objective measures, and it has been suggested that it may be a risk factor for cognitive impairment and possibly dementia^15^. It has been hypothesized that chronic physical conditions may lead to cognitive decline via atherosclerosis, microvascular changes, and inflammatory processes^16^, and these factors may cumulatively amplify the risk for cognitive decline especially in individuals with multimorbidity. However, there is limited evidence on the association between multimorbidity and SCC. This is an important omission because SCC can be a marker of cognitive decline even in the presence of normal objective cognition, while it can serve as a rapid clinical assessment of cognitive function as it does not require burdensome neuropsychological testing^2^.

The first study on multimorbidity and SCC was conducted in 2011 and included more than 15000 persons aged 55 years and over from the Netherlands^8^. This study found a positive association between multimorbidity and SCC, and this association was modified by age, with the relationship between multimorbidity and SCC being stronger in people aged 55–69 years than in those aged 70 years and over. These findings were later corroborated in two other population-based studies from Sweden and Spain^9,10^. In the study conducted in Spain (N = 1342 elderly people), the prevalence of subjective memory complaints increased from 20.8% in participants without any medical condition to 34.1% in those with four or more medical conditions^10^. The main limitations of these three studies are that they included older adults only, despite the fact that SCC is also known to be frequent in younger ages^17^ and that the prevalence of multimorbidity is not negligible even in younger adults^18^. For example, a large community-based study showed that almost 30% of people aged 25–35 years considered themselves as being forgetful^17^, while the prevalence of multimorbidity in people aged 25–44 years has been reported to be 9.3%^18^. Furthermore, previous research investigating the association between cognitive complaints and single physical diseases among young adults used clinical samples (e.g., patients with epilepsy, cancer, schizophrenia) and there are no studies on this association in young people using community-based samples^3,4^. Another important limitation is that none of these studies were nationally representative. This severely limits the generalizability of the previous studies focusing on the multimorbidity-SCC relationship. Furthermore, to the best of our knowledge, there are no studies that aimed to identify potential influential factors in the association between multimorbidity and SCC. Several variables such as obesity^19,20^, smoking^21,22^, alcohol use^23,24^, stress and stressful life events^25–27^, depression^28,29^, anxiety^13,30^ or sleep problems^31,32^ are known to be associated with both multimorbidity and cognitive impairment, and might a play major role in this relationship.

Therefore, the goal of the present study using nationally representative, community-based data from the UK was to examine the association between multimorbidity and SCC, and to quantify the extent to which a broad range of mainly psychological and behavioral factors explain this relationship. Given the high prevalence of SCC in the UK^3^, and the upward trend in multimorbidity mainly associated with increased life expectancy in this setting^33^, a deeper understanding of the multimorbidity-SCC association may provide important information for the prevention and management of cognitive decline and possibly dementia. In particular, since the pathophysiological processes of dementia may begin many years prior to diagnosis^34^, there is the need to focus not only on older populations, but younger populations.

## Results

### Sample characteristics

This nationally representative study included 7399 participants aged ≥16 years without a doctor’s diagnosis of dementia or Alzheimer’s disease. The mean (SD) age of the population was 46.3 (18.6) years and 48.6% were men. The prevalence (95% CI) of multimorbidity, subjective concentration complaints and subjective memory complaints were 35.1% (33.9–36.4%), 22.0% (20.9–23.2%) and 29.9% (28.7–31.1%), respectively. The sample characteristics are reported in Table 1. Compared to people without multimorbidity, people with this condition were significantly more likely to be women, older and British White, while they also had higher mean number of stressful life events, and higher prevalence of no qualification, low income, obesity, smoking, depression, any anxiety disorder and sleep problems.

**Table 1:** Characteristics of individuals included in this study (overall and by physical multimorbidity status).

Characteristics	Category	Overall	Physical multimorbidity		P-value^a^
			Absent	Present	
Sex	Male	48.6	51.6	42.9	<0.001
	Female	51.4	48.4	57.1	
Age (years)	16–44	50.0	61.4	29.1	<0.001
	45–64	31.0	26.9	38.5	
	≥65	19.0	11.7	32.4	
British White	No	14.9	17.0	10.9	<0.001
	Yes	85.1	83.0	89.1	
Qualification	No	23.9	19.7	31.8	<0.001
	Yes	76.1	80.3	68.2	
Income	High	35.8	39.2	29.8	<0.001
	Middle	32.6	32.0	33.8	
	Low	31.6	28.8	36.4	
Obesity	No	82.3	86.3	75.1	<0.001
	Yes	17.7	13.7	24.9	
Smoking status	Never	34.8	36.7	31.4	<0.001
	Quit/Current	65.2	63.3	68.6	
Alcohol dependence	No	92.3	91.5	93.8	0.002
	Yes	7.7	8.5	6.2	
Perceived stress	No	39.4	39.0	40.1	0.398
	Yes	60.6	61.0	59.9	
Number of stressful life events	Mean (SD)	3.4 (2.4)	3.0 (2.2)	4.2 (2.7)	<0.001
Depression	No	97.0	98.0	95.1	<0.001
	Yes	3.0	2.0	4.9	
Any anxiety disorder	No	93.3	95.3	89.4	<0.001
	Yes	6.7	4.7	10.6	
Sleep problems	No	57.6	63.9	46.0	<0.001
	Yes	42.4	36.1	54.0	

Physical multimorbidity was defined as ≥2 physical conditions.

^a^P-values were based on Chi-squared tests except for the number of stressful life events (t-test).

### Prevalence of SCC by number of chronic conditions

**Figure 1:**
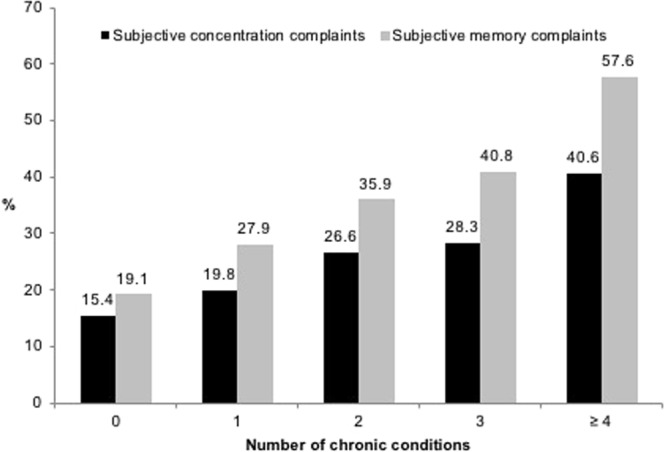
Prevalence of subjective concentration and memory complaints by number of chronic physical conditions. Subjective concentration complaints were assessed with the following question: “In the past month, have you had any problems with concentrating on what you were doing?”. Subjective memory complaints were assessed with the following question: “Have you noticed any problems with forgetting things in the past month?”.

The prevalence of SCC increased with increasing number of chronic conditions with the prevalence ranging from 15.4% in individuals with no chronic conditions to 40.6% in those with ≥4 chronic conditions for subjective concentration complaints and from 19.1% to 57.6% for subjective memory complaints (Fig. 1).

### Prevalence of SCC by presence of physical disease and multimorbidity

The presence of SCC was positively and significantly associated with each of the 20 physical diseases except cancer, diabetes and stroke for subjective concentration complaints and only stroke for subjective memory complaints (Table 2). The OR was particularly large (i.e., OR > 3) for epilepsy/fits and liver problems for subjective concentration complaints, while this was the case for bladder problems/incontinence and epilepsy/fits in terms of subjective memory complaints. Multimorbidity was associated with higher prevalence of subjective concentration (30.7% vs. 17.3%) and memory complaints (42.8% vs. 22.9%) compared to no multimorbidity. The results of the tetrachoric correlation for individual physical diseases by age group are displayed in Appendix 1. There was a stronger tendency for hypertension and diabetes to co-occur or for allergy to co-exist with asthma among the younger age groups. There were fewer distinct patterns in the oldest age group with the strongest correlation being observed between asthma and bronchitis or cataract and epilepsy.

**Table 2:** Prevalence of subjective concentration and memory complaints by physical disease.

Physical disease	Category	Subjective concentration complaints	P-value^a^	OR (95% CI)	Subjective memory complaints	P-value^a^	OR (95% CI)
Allergy	No	21.2	<0.001	1.61 [1.29, 2.03]	29.1	<0.001	1.53 [1.29, 1.82]
	Yes	30.3			38.6		
Arthritis	No	21.0	<0.001	1.54 [1.32, 1.81]	27.9	<0.001	2.07 [1.80, 2.37]
	Yes	29.1			44.4		
Asthma	No	21.1	<0.001	1.72 [1.41, 2.09]	29.0	<0.001	1.60 [1.34, 1.91]
	Yes	31.5			39.5		
Bladder problems/incontinence	No	21.4	<0.001	2.38 [1.85, 3.05]	29.0	<0.001	3.20 [2.51, 4.10]
	Yes	39.3			56.7		
Bone, back, joint or muscle problems	No	19.2	<0.001	1.97 [1.73, 2.24]	26.1	<0.001	2.17 [1.92, 2.46]
	Yes	31.9			43.4		
Bowel/colon problems	No	21.5	<0.001	1.75 [1.37, 2.24]	29.2	<0.001	1.90 [1.55, 2.34]
	Yes	32.3			43.9		
Bronchitis/emphysema	No	21.8	0.002	1.61 [1.19, 2.19]	29.4	<0.001	2.44 [1.82, 3.26]
	Yes	31.0			50.4		
Cancer	No	22.0	0.611	1.12 [0.72, 1.76]	29.7	0.005	1.89 [1.21, 2.95]
	Yes	24.0			44.4		
Cataracts/eyesight problems	No	21.3	<0.001	1.29 [1.11, 1.49]	28.4	<0.001	1.58 [1.38, 1.81]
	Yes	25.9			38.5		
Diabetes	No	21.8	0.086	1.26 [0.97, 1.63]	29.4	<0.001	1.65 [1.31, 2.06]
	Yes	26.0			40.7		
Ear/hear problems	No	21.6	0.007	1.32 [1.08, 1.61]	28.8	<0.001	1.94 [1.59, 2.36]
	Yes	26.7			44.0		
Epilepsy/fits	No	21.8	<0.001	3.43 [1.78, 6.61]	29.8	<0.001	3.12 [1.62, 6.03]
	Yes	48.9			56.9		
Heart attack/angina	No	21.7	<0.001	1.79 [1.31, 2.45]	29.4	<0.001	2.63 [1.97, 3.50]
	Yes	33.2			52.2		
High blood pressure	No	21.5	0.046	1.16 [1.00, 1.35]	28.3	<0.001	1.55 [1.35, 1.78]
	Yes	24.2			37.9		
Infectious disease	No	21.9	0.033	2.14 [1.05, 4.39]	29.8	0.007	2.75 [1.28, 5.88]
	Yes	37.6			53.8		
Liver problems	No	21.8	<0.001	3.61 [2.15, 6.06]	29.7	0.001	2.37 [1.40, 4.01]
	Yes	50.1			50.1		
Migraine or frequent headaches	No	20.4	<0.001	2.66 [2.19, 3.24]	28.1	<0.001	2.61 [2.16, 3.16]
	Yes	40.5			50.5		
Skin problems	No	20.8	<0.001	1.80 [1.50, 2.16]	28.7	<0.001	1.68 [1.40, 2.00]
	Yes	32.2			40.3		
Stomach ulcer or other digestive problems	No	21.2	<0.001	2.07 [1.66, 2.57]	28.8	<0.001	2.34 [1.92, 2.84]
	Yes	35.7			48.6		
Stroke	No	22.0	0.998	1.00 [0.46, 2.18]	29.8	0.110	1.70 [0.88, 3.27]
	Yes	22.0			41.9		

Abbreviations: OR Odds ratio; CI Confidence interval.

Subjective concentration complaints were assessed with the following question: “In the past month, have you had any problems with concentrating on what you were doing?”. Subjective memory complaints were assessed with the following question: “Have you noticed any problems with forgetting things in the past month?”.

^a^P-values were based on Chi-squared tests.

### Association between multimorbidity and SCC estimated by multivariable logistic regression analyses

After adjustment for sociodemographic factors, physical multimorbidity was associated with higher odds for both subjective concentration (OR = 2.58; 95% CI = 2.25–2.96) and memory complaints (OR = 2.34; 95% CI = 2.08–2.62) in the total sample (Table 3). Similar associations were found in the different age groups with ORs ranging from 2.48 to 2.77 for subjective concentration complaints and from 2.12 to 2.40 for subjective memory complaints.

**Table 3:** The association of physical multimorbidity (independent variable) with subjective concentration and memory complaints (dependent variables) estimated by multivariable logistic regression.

	Overall		Aged 16–44 years		Aged 45–64 years		Aged ≥65 years	
	OR	95% CI	OR	95% CI	OR	95% CI	OR	95% CI
SCC	2.58	[2.25, 2.96]	2.48	[2.02, 3.06]	2.77	[2.20, 3.50]	2.57	[1.90, 3.47]
SMC	2.34	[2.08, 2.62]	2.40	[1.91, 3.00]	2.39	[1.97, 2.89]	2.12	[1.67, 2.68]

Abbreviations: OR Odds ratio; CI Confidence interval; SCC Subjective concentration complaints; SMC Subjective memory complaints.

Physical multimorbidity was defined as ≥2 physical conditions.

Subjective concentration complaints were assessed with the following question: “In the past month, have you had any problems with concentrating on what you were doing?”. Subjective memory complaints were assessed with the following question: “Have you noticed any problems with forgetting things in the past month?”.

Models were adjusted for sex, age, ethnicity, qualification and income.

All p-values were lower than 0.001.

### Mediation analysis

Table 4 shows the extent to which the association between multimorbidity and SCC is explained by potential influential factors. Sleep problems explained the largest proportion of the multimorbidity-SCC association, with the mediated percentage being 22.8% for subjective concentration complaints and 20.6% for subjective memory complaints. The two other major influential factors were stressful life events (concentration: 22.1%; memory: 20.1%) and any anxiety disorder (14.9% and 14.4%, respectively). Collectively, the eight influential factors explained 47.5% and 43.3% of the relationship of multimorbidity with subjective concentration and memory complaints, respectively.

**Table 4:** Contribution of potential influential factors in the association of physical multimorbidity with subjective concentration and memory complaints.

	Total effect		Direct effect		Indirect effect		
	OR [95% CI]	P-value	OR [95% CI]	P-value	OR [95% CI]	P-value	%Mediated^a^
***Subjective concentration complaints***							
Obesity	2.58 [2.25, 2.96]	<0.001	2.57 [2.24, 2.95]	<0.001	1.00 [0.99, 1.02]	0.708	NA
Smoking	2.58 [2.26, 2.95]	<0.001	2.56 [2.24, 2.93]	<0.001	1.01 [1.00, 1.02]	0.047	0.8
Alcohol dependence	2.60 [2.27, 2.97]	<0.001	2.57 [2.25, 2.94]	<0.001	1.01 [1.00, 1.02]	0.130	NA
Number of stressful life events	2.60 [2.26, 2.98]	<0.001	2.10 [1.83, 2.41]	<0.001	1.23 [1.19, 1.29]	<0.001	22.1
Perceived stress	2.67 [2.32, 3.06]	<0.001	2.45 [2.13, 2.81]	<0.001	1.09 [1.06, 1.12]	<0.001	8.8
Depression	2.67 [2.32, 3.07]	<0.001	2.44 [2.13, 2.81]	<0.001	1.09 [1.06, 1.12]	<0.001	8.9
Any anxiety disorder	2.64 [2.30, 3.03]	<0.001	2.29 [1.99, 2.62]	<0.001	1.16 [1.12, 1.20]	<0.001	14.9
Sleep problems	2.74 [2.39, 3.15]	<0.001	2.18 [1.90, 2.50]	<0.001	1.26 [1.21, 1.31]	<0.001	22.8
Total	2.88 [2.47, 3.36]	<0.001	1.74 [1.50, 2.03]	<0.001	1.65 [1.54, 1.78]	<0.001	47.5
***Subjective memory complaints***							
Obesity	2.39 [2.13, 2.69]	<0.001	2.37 [2.11, 2.67]	<0.001	1.01 [0.99, 1.02]	0.302	NA
Smoking	2.34 [2.09, 2.63]	<0.001	2.31 [2.06, 2.60]	<0.001	1.01 [1.00, 1.02]	0.010	1.5
Alcohol dependence	2.35 [2.09, 2.64]	<0.001	2.33 [2.07, 2.62]	<0.001	1.01 [1.00, 1.02]	0.133	NA
Number of stressful life events	2.37 [2.11, 2.67]	<0.001	1.99 [1.77, 2.25]	<0.001	1.19 [1.15, 1.23]	<0.001	20.1
Perceived stress	2.38 [2.11, 2.68]	<0.001	2.23 [1.98, 2.51]	<0.001	1.07 [1.05, 1.09]	<0.001	7.6
Depression	2.41 [2.14, 2.71]	<0.001	2.23 [1.98, 2.51]	<0.001	1.08 [1.05, 1.11]	<0.001	8.7
Any anxiety disorder	2.41 [2.14, 2.71]	<0.001	2.12 [1.88, 2.39]	<0.001	1.13 [1.10, 1.17]	<0.001	14.4
Sleep problems	2.44 [2.16, 2.76]	<0.001	2.03 [1.80, 2.29]	<0.001	1.20 [1.16, 1.25]	<0.001	20.6
Total	2.65 [2.33, 3.02]	<0.002	1.74 [1.53, 1.98]	<0.001	1.53 [1.43, 1.63]	<0.001	43.3

Abbreviations: OR Odds ratio; CI Confidence interval.

Physical multimorbidity was defined as ≥2 physical conditions.

Subjective concentration complaints were assessed with the following question: “In the past month, have you had any problems with concentrating on what you were doing?”. Subjective memory complaints were assessed with the following question: “Have you noticed any problems with forgetting things in the past month?”.

Model was adjusted for sex, age, ethnicity, qualification and income.

^a^Mediated percentage was only calculated when the indirect effect was significant (P < 0.05).

## Discussion

We found in this large nationally representative study including individuals aged ≥ 16 years from the UK that the prevalence of SCC increased with the number of chronic conditions. Most individual physical diseases were positively associated with SCC. In addition, multimorbidity was independently associated with a 2.58- and 2.34-fold increase in the odds for subjective concentration and memory complaints, respectively. The association was similar between different age groups despite the fact that the underlying multimorbidity pattern may differ by age groups. Finally, sleep problems, stressful life events and any anxiety disorder individually explained approximately 14–23% of the multimorbidity-SCC relationship.

Most of the individual chronic conditions were positively associated with subjective concentration and memory complaints. In the past years, there has been an important literature about the potential impact of these individual chronic conditions on the risks for SCC. In a cross-sectional epidemiological study, impaired vision and hearing were found to be significant predictors of SCC^10^. The authors hypothesized that, as vision and hearing are tightly related to cognitive functions, severe impairment of these senses might favor the development of memory problems. Another study including 188 patients diagnosed with migraine reported a rate of subjective cognitive decline of 44.7% in the sample, and showed that both depression and short sleep duration during weekdays were risk factors for subjective cognitive decline^35^. More recently, a study including 670 adults attending an epilepsy clinic showed that more than 40% of the patients reported subjective cognitive impairment, and that subjective cognitive impairment was strongly impacted by depression, the number of antiepileptic medications and seizure frequency^36^.

Given the previously reported association between the individual chronic conditions and SCC, it is reasonable to assume that individuals with greater numbers of chronic conditions (i.e., multimorbidity) would be at particularly high risk for SCC. However, apart from the contribution of individual physical contributions to this association, it is also possible that multimorbidity *per se* may be an independent risk factor for SCC. First, multimorbidity is positively associated with frailty^37^, while frailty is related to subjective cognitive decline in older adults without dementia^38^. Second, some chronic conditions (e.g., heart disease, cerebrovascular disease) may synergistically accelerate cognitive decline^15^. Third, people with multimorbidity are at an increased risk for polypharmacy^39^, and polypharmacy is a predictor of poorer cognitive capability^40^.

In our study, multimorbidity was associated with higher odds for SCC, and 21–23%, 20–22%, and 14–15% of this association was individually explained by sleep problems, stressful life events and anxiety, respectively. One population-based study including 42116 adults aged ≥50 years from nine countries found that many of the chronic conditions assessed in our study were associated with sleep problems^31^, and that there was a linear dose-dependent association between the number of chronic conditions and sleep problems. Symptoms of the chronic conditions (e.g., nocturnal symptoms in asthma or nocturia in diabetes) and the potential co-existence of sleep-disordered breathing with some chronic conditions (e.g., chronic lung disease, diabetes) might explain the heightened risk for sleep problems in people with chronic conditions^31^. A positive relationship between sleep problems and objective and subjective cognitive function has further been reported^32,41^. The higher likelihood of use of hypnotics and anxiolytics among people with multimorbidity may partly explain our findings. It has been reported that multimorbidity is associated with a 15-fold increase in the odds of receiving hypnotic or anxiolytic drugs^42^, while the negative impact of these medications on cognition is well-known^43^. Finally, it is also possible that self-perception of cognitive abilities is better in “good sleepers” than in “bad sleepers”^32,44^.

Stressful life events and any anxiety disorder were additional influential factors in the relationship between multimorbidity and SCC. A positive association between childhood adversities and multimorbidity in adulthood has been reported^26^, while another study of earthquake survivors showed prospectively that stressful life events were important determinants of future multimorbidity onset^45^. These findings suggest that stressful life events may be a cause rather than a consequence of multimorbidity, and they underline the fact that multimorbidity may be a complex psychosocial concept. Stressful life events have also been shown to increase the risk for self-reported memory problems^25^. The impact of stressful life events on both multimorbidity and SCC might be mediated by dysregulations in the hypothalamic-pituitary-adrenal (HPA) axis^27,46,47^.

Regarding anxiety disorders, elevated anxiety has been reported to be more frequent in individuals with multimorbidity than in those without multimorbidity^30^. Anxiety levels may be elevated in individuals with multimorbidity because of worries of having an illness and difficulties adjusting to the burden of multimorbidity^48^. An analysis of community-dwelling elderly persons with normal physical examination further revealed that subjective memory complaints were associated with anxiety^13^. Previous studies have also shown that SCC may be a reflection of health anxiety^3^, while studies using objective measures of cognitive function showed that anxiety leads to greater cognitive decline, and that this may be due to interferences with cognitive performance and excessively high concentrations of circulating catecholamines and glucocorticoids^49,50^. Interestingly, in our study, depression was not a mediator in the association between multimorbidity and SCC. This was an unexpected finding given the previous reports that depression is highly prevalent in individuals with somatic disorders^51^ and that depression and cognitive impairment are highly comorbid^52^. The reason for this is unclear and future studies are warranted to assess whether our results can be corroborated.

Finally, the fact that SCC was significantly associated with multimorbidity even after adjustment for all the potential influential factors (i.e., significant direct effect) suggests that there are other factors which may explain the multimorbidity-SCC association. These factors might include inflammation, dysregulation of the HPA axis, fatigue, polypharmacy (≥5 drugs taken at the same time) and drugs interactions^9,11^.

Interestingly, we also found that the strength of the multimorbidity-SCC association was similar between the different age groups (16–44, 45–64 and ≥65 years), suggesting that it may be important to focus on multimorbidity throughout the life course as a potential risk factor for SCC and possibly future cognitive decline. This finding is of particular interest because our tetrachoric analysis highlighted important differences in terms of correlations between individual physical diseases by age group, and because previous research has shown that multimorbidity patterns vary with age^53^, suggesting that the effects of multimorbidity on SCC likely involve a wide range of chronic conditions. However, since contradictory results have been obtained in the past, these results must be interpreted with caution. In fact, one study found that there was a significant two-way interaction between physical multimorbidity and age, and that the relationship of multimorbidity with SCC was stronger in participants aged 55–69 years than in those aged ≥70 years^8^. The authors suggested that, this may be due to the fact that younger individuals are professionally active in life, and that they might experience problems related to their forgetfulness more frequently than older individuals.

Based on the present findings, clinicians should be aware that subjective concentration and memory complaints are highly prevalent in individuals with multimorbidity. Screening for SCC among people with multimorbidity may be important at all ages for the prevention of more severe cognitive disorders in this population. In particular, there is burgeoning evidence that intervening in mid-life is crucial^54^, although how cognitive function (especially SCC) among younger adults predicts future cognitive decline is currently largely unknown and should be assessed in future studies. The subjective nature of SCC makes it a much more likely candidate for rapid clinical assessment for cognitive function in individuals with multimorbidity given that SCC can be self-reported and does not require burdensome neuropsychological testing and specially trained staff. Based on our study results, it is possible that sleep problems, stressful life events and anxiety may be underlying factors which contribute to a higher odds for SSC in individuals with multimorbidity. When an individual with multimorbidity is found to have SCC, the clinician can screen for and potentially manage these conditions to improve SCC not only to reduce the risk for future cognitive decline but also to improve clinical outcome and the quality of life of the patient. Finally, in terms of future research, longitudinal studies are warranted to provide further insight into the potentially complex interplay of factors that link multimorbidity and SCC, while based on our findings, we call for future work on the age difference in the multimorbidity-SCC association, the role of depression in this association, and the potential utility of screening for SCC in individuals with physical diseases or multimorbidity to identify individuals at high risk for future cognitive decline and possibly dementia.

The strengths of the study include the large sample size, the use of nationally representative data, and the analysis which allowed for quantification of the extent to which various factors might explain the multimorbidity-SCC relationship. However, the study results should be interpreted in the light of several limitations. First, although we included 20 common physical diseases in our study, this is by no means an exhaustive list of all physical conditions. Thus, the results may have differed if more diseases were included. Second, subjective concentration and memory complaints were assessed with a single question. Thus, it is possible that the association between multimorbidity and SCC observed in our study may have differed with the use of a more complete measure. However, there are no standardized measures to capture SCC, and the use of a single question to assess SCC is common^4^. Furthermore, the prevalence of SCC in our study was within previously reported ranges^3,55,56^. Third, although the accuracy of patients’ self-reports of diagnoses has been previously reported to be satisfactory^57,58^, the use of self-reported data may have biased our findings. Fourth, individuals who were considered to have a level of cognitive impairment severe enough to preclude the possibility to participate in the survey, and those with a self-reported diagnosis of dementia or Alzheimer’s disease were excluded from the analysis. However, owing to the fact that the study was not designed to make clinical diagnoses of dementia, it is possible that the sample does not consist solely of cognitively healthy individuals. Fifth, because the survey did not include the institutionalized and the homeless, our study findings cannot be generalized to these populations, where multimorbidity and SCC may be particularly prevalent. Sixth, the survey response rate was 57% and this may have impacted the present findings. That being said, this rate is similar to previous national surveys^59^. Furthermore, non-responders were likely to have had similar characteristics to responders, as there were only minor differences in the results when using and not using non-response weights, which corrected for a range of sociodemographic and area characteristics. Finally, the design of the present study was cross-sectional, and it was thus not possible to determine causality or temporality in the multimorbidity-SCC relationship. Relatedly, mediation and confounding are identical statistically and can be distinguished only on conceptual grounds^60^. While many of the influential factors assessed in this study can be conceptualized as mediators, it is not possible to determine whether the attenuation in the ORs after the inclusion of the influential factor is due to mediation or confounding in our cross-sectional study. Nonetheless, given that the influential factors in the association between multimorbidity and SCC are largely unknown, we believe that our study provides an important platform for future longitudinal studies to provide more concrete evidence for the establishment of causality.

In conclusion, the prevalence of SCC increased with the number of chronic conditions. The association between multimorbidity and SCC remained significant after adjustment for a variety of factors. Sleep problems, the number of stressful life events and any anxiety disorder explained 14–23% of the relationship. Based on these results, we recommend routine screening of SCC and the management of the potential underlying causes of it in people with multimorbidity as this may lead to reduced risk for future cognitive decline and also improve patient wellbeing or clinical outcomes although more longitudinal and interventional studies are warranted to elucidate the utility of assessing SCC in individuals with multimorbidity.

## Methods

### Study participants

This study used data from 7403 people who participated in the 2007 Adult Psychiatric Morbidity Survey (APMS). Full details of the survey have been published elsewhere^61^. Briefly, this was a nationally representative survey of the English adult population (aged ≥16 years) living in private households. The National Center for Social Research and Leicester University undertook the survey fieldwork in October 2006 to December 2007 using a multistage stratified probability sampling design where the sampling frame consisted of the small user postcode address file (a listing of all postal delivery points), while the primary sampling units were postcode sectors. Participant information was obtained through face-to-face interviews where some of the questionnaire items were self-completed (with the use of a computer). The interviewer was instructed to conduct a short proxy interview if the selected respondent was not capable of taking part in the survey due to health-related illness, stay in hospital, cognitive impairment, or mental incapacity. The data for these proxy interviews were not included in the publically available dataset. Sampling weights were constructed to account for non-response and the probability of being selected so that the sample was representative of the English adult household population. The survey response rate was 57%. Ethical permission for the study was obtained from the Royal Free Hospital and Medical School Research Ethics Committee. All participants provided informed consent before their inclusion. All methods were performed in accordance with the relevant guidelines and regulations.

### Measures

#### Physical multimorbidity

We included data on all 20 physical conditions for which data were available in the APMS^62^. These physical conditions were allergy, arthritis, asthma, bladder problems/incontinence, bone/back/joint/muscle problems, bowel/colon problems, bronchitis/emphysema, cancer, cataract/eyesight problems, diabetes, ear/hearing problems, epilepsy, heart attack/angina, high blood pressure, infectious disease, liver problems, migraine, skin problems, stomach ulcer or other digestive problems, and stroke. These 20 diseases are highly prevalent in the general population and are known to have a high burden at the population level. Participants self-reported whether they ever had pre-specified doctor diagnosed conditions since the age of 16. Specifically, the participant was presented with a card with the names of the health conditions and asked whether he or she ever had any of them since the age of 16. For each health condition, the participant was also asked whether a doctor or health professional diagnosed the condition, and whether the participant had this health condition in the past 12 months. We only included health conditions which were diagnosed by a doctor or a health professional, and were present in the past 12 months. The number of physical diseases was summed and categorized as 0, 1, 2, 3, and ≥4. Multimorbidity was defined as the presence of two or more physical diseases^62^.

#### Subjective cognitive complaints

SCC included subjective concentration complaints and subjective memory complaints. In line with a previous APMS study^4^, subjective concentration and memory complaints were assessed with two questions: “In the past month, have you had any problems with concentrating on what you were doing?” (concentration) and “Have you noticed any problems with forgetting things in the past month?” (memory) with “yes” and “no” answer options. Two separate dichotomous variables were created for concentration and memory based on these answer options.

#### Influential variables

The influential factors (obesity^19,20^, smoking^21,22^, alcohol use^23,24^, stress and stressful life events^25–27^, depression^28,29^, anxiety^13,30^, and sleep problems^31,32^) were selected based on their previously reported associations with SCC and multimorbidity.

Obesity: Body mass index (BMI) was calculated as weight in kilograms divided by height in meters squared based on self-reported weight and height^62^. Using the standard WHO definition, obesity was defined as BMI ≥ 30 kg/m^2^, and BMI < 30 kg/m^2^ was considered no obesity.

Smoking status: Smoking was assessed by the question ‘Have you ever smoked a cigarette?’ with answer options “yes” or “no”. Participants were dichotomized as never or current/past smokers^62^.

Alcohol dependence: Excessive alcohol consumption was screened using the Alcohol Use Disorders Identification Test (AUDIT)^63^. Alcohol dependence was assessed with the Severity of Alcohol Dependence Questionnaire (SADQ-C) in participants with an AUDIT score of 10 or above^64^. Scores of four or above indicated alcohol dependence in the past six months.

Stressful life events: Eighteen items were used to assess different stressful life events (e.g., serious illness, death of an immediate family member or major financial crises)^65^. Therefore, the scale ranged from 0 to 18.

Perceived stress: Participants were asked if their tasks at home and at work were stressful^66^. Answers ranged from 0 “not at all” to 3 “most of the time”. Stress was then dichotomized into “not at all” or “occasionally”, “usually” and “most of the time”.

Common mental disorders: Common mental disorders were assessed using the Clinical Interview Schedule Revised (CIS-R)^67,68^, and referred to depressive episode and anxiety disorders (generalized anxiety disorder, panic disorder, phobia and obsessive-compulsive disorder) in the prior week^69^.

Sleep problems: Two questions with “yes” and “no” answer options were used to assess sleep problems: “In the past month, have you been having problems with trying to get to sleep or with getting back to sleep if you woke up or were woken up?” (sleeping less than usual) and “Has sleeping more than you usually do been a problem for you in the past month?” (sleeping more than usual). Participants were considered as having sleep problems if they reported sleeping less or more than usual^70^.

### Control variables

#### Sociodemographic variables

These included sex, age [16–44 (young adults), 45–64 (middle-aged adults) and ≥65 years (older adults)], ethnicity (British White: yes or no), qualification (yes or no) and income (sex equivalized income tertiles; high ≥£29,826, middle £14,057–<£29,826 and low <£14,057)^62^. Participants were considered qualified if their highest educational qualification was one of the following: degree level; teaching, Higher National Diploma (HND) or nursing; A-level; General Certificate of Secondary Education (GCSE) or equivalent; and foreign or other qualifications.

### Statistical analyses

We deleted the four individuals who self-reported a doctor’s diagnosis of dementia or Alzheimer’s disease to focus on a cognitively healthy sample. Differences between groups were tested with chi-squared tests for categorical variables and Student’s *t*-tests for continuous variables (i.e., prevalence of each sample characteristics among those with and without multimorbidity, and prevalence of SCC among those with and without the individual physical diseases). Since previous research has found that multimorbidity patterns may vary with age^53^, tetrachoric correlations between each chronic condition were calculated by age groups. The association between individual physical diseases and SCC was estimated by unadjusted logistic regression analysis. The association between multimorbidity (explanatory variable) and subjective concentration and memory complaints (outcome variables) was assessed by multivariable logistic regression. The model was adjusted for sex, age, ethnicity, qualification and income. As more than 20% of the participants had missing income information, a missing category for this variable was included in all regression analyses in order to avoid excluding a large number of respondents from the analysis. All variables were included in the regression models as categorical variables with the exception of the number of stressful life events (continuous variable). All categorical variables were dichotomous variables except age and income, which had more than two categories. Age stratified analyses (16–44, 45–64 and ≥65 years) were also conducted as the association between multimorbidity and SCC may differ by age^71^. In order to assess the influence of multicollinearity, the variance inflation factor (VIF) value was calculated for each independent variable. The highest VIF was 1.60, which is much lower than the commonly used-cut off of 10^72^, indicating that multicollinearity was unlikely to be a problem. Using the ado-command *svylogitgof* that allows to account for the complex survey design^73^, model fit was evaluated with the F-adjusted mean residual test, and this test suggested that final models were a good fit for the data.

Finally, mediation analysis was conducted to assess the specific contribution of the different potential influential factors in the multimorbidity-SCC relationship. We used the *khb* (Karlson Holm Breen) command in Stata for this purpose^74^. This method can be applied in logistic regression models and decomposes the total effect of a variable into direct and indirect effects. Using this method, the percentage of the main association explained by the influential factor can also be calculated (mediated percentage). The mediated percentage is the percent attenuation in the log odds for the association between multimorbidity and SCC after the influential factor is included in the model^74^. The mediation analysis controlled for sex, age, ethnicity, qualification and income. All influential factors were included individually in the model with the exception of the final model where all influential factors were included simultaneously.

We used the Stata *svy* command that allows for the use of clustered data modified by probability weights. Results from the logistic regression analyses are presented as odds ratios (ORs) with 95% confidence intervals (CIs). The level of statistical significance was set at p-value < 0.05. All analyses were performed with Stata version 13.1 (Stata Corp LP, College Station, Texas).

## Supplementary information

Appendix 1. Tetrachoric correlations of physical health conditions by age group

## Data Availability

Data can be accessed via the website of the UK Data Archive. However, the present authors cannot directly share the data and a request must be sent to the UK Data Archive.
