# Supplementary material for: Physical multimorbidity and subjective cognitive complaints among adults in the United Kingdom: a cross-sectional community-based study
Source: Sci Rep. 2019 Aug 27;9:12417. doi: 10.1038/s41598-019-48894-8 (PMC6711967; doi:10.1038/s41598-019-48894-8)
Supplement: Supplementary file 1 — Appendix 1. Tetrachoric correlations of physical health conditions by age group [file 41598_2019_48894_MOESM1_ESM.docx]

Physical multimorbidity and subjective cognitive complaints among adults in the United Kingdom: a cross-sectional community-based study

Louis Jacob, PhD; Josep Maria Haro, MD-PhD; Ai Koyanagi, MD-PhD

**Appendix 1.** Tetrachoric correlations of physical health conditions by age group

| 16-44 years | | | | | | | | | | | | | | | | | | | | |
| --- | --- | --- | --- | --- | --- | --- | --- | --- | --- | --- | --- | --- | --- | --- | --- | --- | --- | --- | --- | --- |
|  | 1 | 2 | 3 | 4 | 5 | 6 | 7 | 8 | 9 | 10 | 11 | 12 | 13 | 14 | 15 | 16 | 17 | 18 | 19 | 20 |
| 1 | 1.00 |  |  |  |  |  |  |  |  |  |  |  |  |  |  |  |  |  |  |  |
| 2 | 0.06 | 1.00 |  |  |  |  |  |  |  |  |  |  |  |  |  |  |  |  |  |  |
| 3 | 0.40* | 0.15* | 1.00 |  |  |  |  |  |  |  |  |  |  |  |  |  |  |  |  |  |
| 4 | 0.17 | -0.01 | 0.18 | 1.00 |  |  |  |  |  |  |  |  |  |  |  |  |  |  |  |  |
| 5 | 0.16* | 0.45* | 0.17* | 0.12 | 1.00 |  |  |  |  |  |  |  |  |  |  |  |  |  |  |  |
| 6 | 0.15* | 0.16 | 0.18* | 0.31* | 0.20* | 1.00 |  |  |  |  |  |  |  |  |  |  |  |  |  |  |
| 7 | 0.34* | 0.42* | 0.38* | NA | 0.27* | 0.23 | 1.00 |  |  |  |  |  |  |  |  |  |  |  |  |  |
| 8 | NA | NA | 0.11 | NA | NA | 0.27 | NA | 1.00 |  |  |  |  |  |  |  |  |  |  |  |  |
| 9 | 0.12* | 0.14 | 0.09 | 0.05 | 0.13* | 0.15* | 0.30* | NA | 1.00 |  |  |  |  |  |  |  |  |  |  |  |
| 10 | NA | 0.33* | 0.01 | NA | -0.13 | NA | NA | NA | 0.30* | 1.00 |  |  |  |  |  |  |  |  |  |  |
| 11 | 0.15* | 0.31* | 0.09 | 0.18 | 0.18* | 0.21* | 0.40* | NA | 0.15* | 0.15 | 1.00 |  |  |  |  |  |  |  |  |  |
| 12 | 0.00 | 0.25 | 0.29* | NA | 0.03 | NA | 0.28 | NA | 0.00 | NA | 0.32* | 1.00 |  |  |  |  |  |  |  |  |
| 13 | NA | NA | NA | NA | 0.15 | NA | NA | NA | NA | NA | NA | NA | 1.00 |  |  |  |  |  |  |  |
| 14 | 0.01 | 0.09 | 0.05 | 0.08 | 0.10 | 0.12 | 0.17 | NA | 0.09 | 0.44* | 0.14 | 0.00 | 0.59* | 1.00 |  |  |  |  |  |  |
| 15 | 0.03 | NA | 0.03 | 0.23 | -0.10 | 0.21 | NA | NA | 0.25* | NA | 0.11 | NA | NA | 0.02 | 1.00 |  |  |  |  |  |
| 16 | 0.15 | 0.10 | 0.16 | 0.35* | 0.02 | 0.35* | NA | 0.51* | -0.01 | NA | NA | NA | 0.63* | 0.35* | 0.33 | 1.00 |  |  |  |  |
| 17 | 0.14* | 0.24* | 0.19* | 0.17 | 0.23* | 0.28* | 0.41* | NA | 0.15* | -0.08 | 0.10 | 0.16 | NA | 0.25* | 0.02 | 0.08 | 1.00 |  |  |  |
| 18 | 0.37* | 0.15* | 0.21* | 0.17 | 0.05 | 0.18* | 0.15 | 0.10 | 0.06 | 0.00 | 0.18* | 0.09 | NA | 0.07 | 0.25* | 0.21 | 0.13* | 1.00 |  |  |
| 19 | 0.33* | 0.13 | 0.12 | 0.15 | 0.20* | 0.36* | 0.37* | NA | 0.18* | 0.21 | 0.25* | 0.20 | NA | 0.21* | 0.22 | 0.28* | 0.31* | 0.17* | 1.00 |  |
| 20 | 0.37 | 0.56 | 0.38 | NA | 0.27 | NA | NA | NA | NA | NA | NA | NA | NA | NA | NA | NA | NA | NA | 0.52 | 1.00 |
| 45-64 years | | | | | | | | | | | | | | | | | | | | |
| 1 | 1.00 |  |  |  |  |  |  |  |  |  |  |  |  |  |  |  |  |  |  |  |
| 2 | 0.13* | 1.00 |  |  |  |  |  |  |  |  |  |  |  |  |  |  |  |  |  |  |
| 3 | 0.39* | 0.20* | 1.00 |  |  |  |  |  |  |  |  |  |  |  |  |  |  |  |  |  |
| 4 | 0.22* | 0.29* | 0.24* | 1.00 |  |  |  |  |  |  |  |  |  |  |  |  |  |  |  |  |
| 5 | 0.22* | 0.37* | 0.15* | 0.32* | 1.00 |  |  |  |  |  |  |  |  |  |  |  |  |  |  |  |
| 6 | 0.08 | 0.18* | 0.18* | 0.41* | 0.25* | 1.00 |  |  |  |  |  |  |  |  |  |  |  |  |  |  |
| 7 | 0.20* | 0.26* | 0.45* | 0.18 | 0.20* | 0.20* | 1.00 |  |  |  |  |  |  |  |  |  |  |  |  |  |
| 8 | 0.08 | 0.03 | -0.11 | 0.27* | 0.02 | 0.14 | -0.01 | 1.00 |  |  |  |  |  |  |  |  |  |  |  |  |
| 9 | 0.03 | 0.04 | 0.04 | 0.17* | 0.19* | 0.08 | 0.17* | 0.10 | 1.00 |  |  |  |  |  |  |  |  |  |  |  |
| 10 | -0.04 | 0.11 | -0.01 | 0.06 | 0.06 | -0.20* | 0.15 | 0.04 | 0.18* | 1.00 |  |  |  |  |  |  |  |  |  |  |
| 11 | 0.09 | 0.24* | 0.14* | 0.28* | 0.12* | 0.11 | 0.05 | -0.10 | 0.16* | 0.00 | 1.00 |  |  |  |  |  |  |  |  |  |
| 12 | NA | -0.04 | -0.09 | NA | 0.02 | -0.05 | NA | NA | 0.15 | -0.04 | 0.14 | 1.00 |  |  |  |  |  |  |  |  |
| 13 | -0.21 | 0.29* | 0.13 | 0.16 | 0.05 | -0.02 | 0.24* | NA | -0.01 | 0.39 | 0.07 | 0.10 | 1.00 |  |  |  |  |  |  |  |
| 14 | 0.03 | 0.17* | 0.08 | 0.16* | 0.05 | 0.08 | 0.09 | 0.04 | 0.04 | 0.46* | 0.15* | -0.11 | 0.31* | 1.00 |  |  |  |  |  |  |
| 15 | 0.40* | 0.30* | 0.20 | 0.11 | 0.41* | 0.14 | 0.16 | 0.39* | 0.25* | 0.16* | NA | 0.50* | NA | -0.04 | 1.00 |  |  |  |  |  |
| 16 | -0.05 | 0.27* | 0.16 | 0.34* | 0.02 | 0.26* | 0.04 | 0.26 | 0.08 | 0.10 | 0.12 | 0.23 | 0.40* | 0.28* | NA | 1.00 |  |  |  |  |
| 17 | 0.08 | 0.28* | 0.06 | 0.28* | 0.29* | 0.20* | 0.19* | 0.10 | 0.16* | -0.03 | 0.02 | 0.17 | 0.02 | 0.03 | 0.24 | 0.07 | 1.00 |  |  |  |
| 18 | 0.40* | 0.16* | 0.25* | 0.14 | 0.13* | 0.27* | 0.15 | 0.24* | 0.09 | 0.03 | 0.04 | 0.06 | 0.08 | 0.09* | 0.28* | -0.11 | 0.10 | 1.00 |  |  |
| 19 | 0.08 | 0.26* | 0.30* | 0.24* | 0.33* | 0.32* | 0.32* | -0.21 | 0.12* | 0.03 | 0.09 | 0.22 | 0.26* | 0.09 | 0.21 | 0.36* | 0.21* | 0.15* | 1.00 |  |
| 20 | 0.07 | NA | 0.08 | NA | -0.18 | NA | 0.28 | NA | -0.07 | NA | NA | 0.45 | NA | 0.02 | NA | 0.39 | 0.05 | 0.02 | NA | 1.00 |
| ≥65 years | | | | | | | | | | | | | | | | | | | | |
| 1 | 1.00 |  |  |  |  |  |  |  |  |  |  |  |  |  |  |  |  |  |  |  |
| 2 | 0.23* | 1.00 |  |  |  |  |  |  |  |  |  |  |  |  |  |  |  |  |  |  |
| 3 | 0.25* | 0.19* | 1.00 |  |  |  |  |  |  |  |  |  |  |  |  |  |  |  |  |  |
| 4 | 0.09 | 0.22* | 0.13* | 1.00 |  |  |  |  |  |  |  |  |  |  |  |  |  |  |  |  |
| 5 | 0.18* | 0.39* | 0.04 | 0.26* | 1.00 |  |  |  |  |  |  |  |  |  |  |  |  |  |  |  |
| 6 | 0.27* | 0.16* | 0.12* | 0.28* | 0.25* | 1.00 |  |  |  |  |  |  |  |  |  |  |  |  |  |  |
| 7 | 0.25* | 0.14* | 0.49* | 0.11 | 0.12* | 0.07 | 1.00 |  |  |  |  |  |  |  |  |  |  |  |  |  |
| 8 | -0.04 | 0.04 | 0.10 | 0.20* | 0.12 | 0.14 | -0.01 | 1.00 |  |  |  |  |  |  |  |  |  |  |  |  |
| 9 | 0.23* | 0.24* | 0.13* | 0.17* | 0.21* | 0.11* | 0.15* | 0.07 | 1.00 |  |  |  |  |  |  |  |  |  |  |  |
| 10 | 0.08 | 0.14* | 0.10 | 0.11 | 0.06 | 0.13 | 0.03 | 0.06 | 0.14* | 1.00 |  |  |  |  |  |  |  |  |  |  |
| 11 | 0.17* | 0.14* | 0.14* | 0.19* | 0.13* | 0.23* | 0.12* | 0.14 | 0.23* | -0.01 | 1.00 |  |  |  |  |  |  |  |  |  |
| 12 | NA | -0.01 | NA | NA | 0.06 | NA | 0.35 | NA | 1.00* | 0.26 | 0.16 | 1.00 |  |  |  |  |  |  |  |  |
| 13 | 0.20* | 0.21* | 0.03 | 0.23* | 0.21* | 0.22* | 0.15* | 0.15 | 0.21* | 0.29* | 0.19* | 0.32 | 1.00 |  |  |  |  |  |  |  |
| 14 | 0.06 | 0.13* | 0.10* | 0.09 | 0.16* | 0.08 | 0.04 | 0.01 | 0.08* | 0.22* | 0.05 | 0.21 | 0.20* | 1.00 |  |  |  |  |  |  |
| 15 | NA | 0.13 | 0.21 | 0.22 | 0.19 | NA | NA | NA | NA | NA | 0.09 | NA | NA | -0.13 | 1.00 |  |  |  |  |  |
| 16 | 0.04 | -0.01 | 0.18 | 0.20 | 0.13 | 0.22 | 0.16 | 0.12 | 0.00 | 0.37* | 0.12 | 0.64* | 0.12 | 0.12 | NA | 1.00 |  |  |  |  |
| 17 | 0.12 | 0.16* | -0.03 | 0.08 | 0.23* | 0.16* | 0.08 | 0.04 | 0.06 | -0.17 | 0.08 | NA | 0.03 | 0.03 | NA | NA | 1.00 |  |  |  |
| 18 | 0.29* | 0.15* | 0.18* | 0.15* | 0.20* | 0.10 | 0.16* | 0.07 | 0.15* | -0.02 | 0.15* | 0.29 | 0.16* | 0.03 | NA | NA | 0.15 | 1.00 |  |  |
| 19 | 0.19* | 0.23* | 0.15* | 0.26* | 0.36* | 0.36* | 0.15* | -0.06 | 0.17* | -0.03 | 0.05 | NA | 0.11 | 0.13* | 0.21 | 0.18 | 0.22* | 0.10 | 1.00 |  |
| 20 | 0.20 | 0.03 | 0.31* | -0.07 | -0.16 | 0.17 | 0.09 | 0.21 | 0.19* | 0.10 | 0.11 | NA | 0.18 | 0.32* | NA | 0.25 | -0.08 | -0.07 | 0.07 | 1.00 |

*P-value lower than 0.05.

Tetrachoric correlation was not calculated when there were cells with 0 (NA).

1=allergy, 2=arthritis, 3=asthma, 4=bladder problems/incontinence, 5=bone, back, joint or muscle problems, 6=bowel/colon problems, 7=bronchitis/emphysema, 8=cancer, 9=cataract/eyesight problems, 10=diabetes, 11=ear/hearing problems, 12=epilepsy/fits, 13=heart attack/angina, 14=high blood pressure, 15=infectious disease, 16=liver problems, 17=migraine or frequent headaches, 18=skin problems, 19=stomach ulcer or other digestive problems, and 20=stroke.
